# Quercetin Treatment Ameliorates Systemic Oxidative Stress in Cirrhotic Rats

**DOI:** 10.5402/2011/604071

**Published:** 2011-08-15

**Authors:** Emanuelle Kerber Vieira, Silvia Bona, Fábio Cangeri Di Naso, Marilene Porawski, Juliana Tieppo, Norma Possa Marroni

**Affiliations:** ^1^Laboratory of Experimental Hepatology and Physiology, Hospital de Clínicas de Porto Alegre, Universidade Federal do Rio Grande do Sul, 90050170 Porto Alegre, RS, Brazil; ^2^Programa de Pós-Graduação em Genética e Toxicologia Aplicada, Universidade Luterana do Brasil, 92120-015 Canoas, RS, Brazil; ^3^Universidade Federal de Ciências da Saúde de Porto Alegre (UFCSPA), 90050-170 Porto Alegre, RS, Brazil; ^3^Laboratório de Estresse Oxidativo e Antioxidantes (ULBRA), Avenida Farroupilha 8001, Bairro São José, 92425-900 Canoas, RS, Brazil

## Abstract

Our aim was to investigate whether the antioxidant quercetin protects against liver injury and ameliorates the systemic oxidative stress in rats with common bile duct ligation. Secondary biliary cirrhosis was induced through 28 days of bile duct obstruction. Animals received quercetin (Q) after 14 days of obstruction. Groups of control (CO) and cirrhotic (CBDL) animals received a daily 50 mg/kg body weight i.p. injection of quercetin (CO + Q; CBDL + Q) or vehicle (CO; CBDL). Quercetin corrected the reduction in superoxide dismutase (SOD), catalase CAT, and glutathione peroxidase GPx activities and prevented the increase of thiobarbituric acid reactive substances (TBARS), aminotransferases, and alkaline phosphatase in cirrhotic animals. Quercetin administration also corrected the reduced total nitrate concentration in the liver and prevented liver fibrosis and necrosis. These effects suggest that quercetin might be a useful agent to preserve liver function and prevent systemic oxidative stress.

## 1. Introduction

Biliary cirrhosis is extrahepatic structure and function and is a potential late complication resulting from prolonged obstruction of extrahepatic biliary pathways. It occurs in less than one tenth of patients with biliary cirrhosis, as described previously [1]. This disease is accompanied by difficulty in bile elimination due to the destruction of hepatic parenchyma and progressive fibrosis [2]. 

 The cytotoxicity of biliary acids is attributed to several mechanisms, including its detergent properties, alterations in intracellular Ca^++^ homeostasis, decreases in ATP, and mitochondrial damage. It has been suggested that alterations in antioxidant mechanisms occur with the pathogenesis of cholestatic hepatic damage, resulting in an imbalance of oxidative and antioxidative processes, which stimulates lipoperoxidation and leads to injuries in several systems [3]. The concentration of bile acids increases in rats after bile duct obstruction, which induces lipid peroxidation, and is probably related to the stimulation of phagocytic activity in polymorphonuclear phagocytes and inflammatory cells [4].

Many experimental studies have reported beneficial effects of antioxidants in cholestasis [5–7]. Intraperitoneal administration of resveratrol maintained antioxidant defenses and reduced liver oxidative damage, ductular proliferation, and fibrosis in bile duct ligated rats [8].

Flavonoids are phenolic phytochemicals that represent essential constituents of the nonenergetic part of the human diet. They are thought to promote optimal health, partly via their antioxidant effects in protecting cellular components against reactive oxygen species (ROS) [9, 10]. Some compounds that have been studied as possible protectors against liver cirrhosis are known for their anti-inflammatory and antioxidant properties. Plants contain numerous polyphenols, which have been shown to reduce inflammation and thereby increase resistance to disease [11]. Quercetin (Q), a polyphenolic flavonoid compound present in large amounts in vegetables, fruits, and tea, exhibits therapeutic potential, including hepatoprotection and the inhibition of liver fibrosis, against many diseases [10, 12, 13]. It contains a number of phenolic hydroxyl groups that have strong antioxidant activity [14, 15]. The average intake varies among people by country but is approximately 23 mg/day; quercetin is predominant at 16 mg/day [14].

By increasing the endogenous antioxidant defenses, flavonoids can modulate the redox state of organisms. The major endogenous antioxidant systems include superoxide dismutase (SOD), catalase (CAT), glutathione reductase (GR), and glutathione peroxidase (GPx), which are essential for the detoxification of lipid peroxides [12, 16].

In cirrhosis patients, the use of blood markers to determine the oxidative stress level and the efficiency of the antioxidant used may qualify the diagnosis and allow for a better survival curve [17]. The purpose of our study was to ascertain whether treatment with quercetin ameliorates the systemic oxidative stress in rats with common bile duct obstruction.

## 2. Materials and Methods

### 2.1. Animals and Experimental Procedures

All studies were performed in accordance with the Guiding Principles for Research Involving Animals at HCPA [18]. Male Wistar rats were obtained from the CREAL (Centro de Reprodução Animal) of the UFRGS, Porto Alegre, Brazil. They were housed in temperature- and humidity-controlled rooms with a 12-hr light-dark cycle and with standard rat chow and free access to tap water. Secondary biliary cirrhosis was induced in animals weighing approximately 250 g by double ligation and division of the common bile duct. The animals were sacrificed after 28 days of obstruction, a time at which there is complete development of both cholestasis and fibrosis and clear establishment of liver biochemical changes [3]. Control animals were sham-operated. Quercetin (Sigma) was resuspended immediately before administration in a 2% tween aqueous solution. Treatment was initiated after 14 days of obstruction. Groups of control (CO) and cirrhotic (CBDL) animals received a daily 50 mg/kg body i.p. injection of quercetin (CO + Q, *n* = 7; CBDL + Q, *n* = 7) or vehicle (CO, *n* = 7; CBDL, *n* = 7). The rats were anaesthetized with 2% xylazine hydrochloride (50 mg/kg) and ketamine hydrochloride (100 mg/kg) administered by i.p. injection. Venous blood was collected in two aliquots from the orbital sinus [19] for the systemic oxidative stress analyses and for analysis of aspartate aminotransferase (AST), alanine aminotransferase (ALT), and alkaline phosphatase (ALP) activities (performed by the Laboratory of Pathology at HCPA-RS, Brazil). The livers were excised, weighed, and divided for histological and biochemical analyses. 

### 2.2. Biochemical Analysis

Plasma was collected from the remaining sample by centrifugation at 2195 ×g for 5 min at 4°C. Erythrocytes were washed with cold saline three times. Hemoglobin content of blood was estimated by the cyanmethemoglobin method described by Drabkin and Austin [20], and the amount of aldehydic products generated by lipid peroxidation in erythrocytes was quantified with a thiobarbituric acid (TBA) reaction. The results were referred to as TBARS [21]. SOD (EC 1.15.1.1) in erythrocytes was assayed according to the protocol of Misra and Fridovich [22]; CAT (EC 1.11.1.6) activity was determined by measuring the exponential disappearance of H_2_O_2_ at 240 nm according to the protocol of Aebi [23]. Analysis of GPx (EC 1.11.1.19) activity was carried out according to the method of Flohe and Guntzler [24]. Protein concentration was measured according to the method of Lowry et al. [25] using serum bovine albumin as standard.

For histological studies, a piece of the liver was trimmed and fixed by immersion in 10% buffered formalin for 24 hr. The obtained blocks were dehydrated in a graded series of ethanol, embedded in paraffin, and stained with hematoxylin and eosin or picrosírius, which mainly stains collagen fibers (performed by the Laboratory of Pathology at HCPA-RS, Brazil).

Frozen liver from each rat was homogenized in ice-cold phosphate buffer (KCl 140 mmol/L and phosphate 20 mmol/L, pH 7.4) and centrifuged at 14,000 ×g for 10 min. Nitric oxide production was measured indirectly using a quantitative colorimetric assay based on the Griess reaction, according to Granger et al. [26]. 

### 2.3. Statistical Analysis

Results were expressed as the mean values ± SEM. The data were compared by analysis of variance (ANOVA); when the analysis indicated a significant difference, the means were compared with the Student Newman-Keuls test. Values were considered significant at *P* < 0.05.

## 3. Results

### 3.1. AST, ALT, and ALP

The cirrhotic group had higher levels of AST, ALT, and ALP compared to the control group. Treatment with quercetin significantly reduced the higher levels of aminotransferases and ALP induced by biliary obstruction (Table 1).

### 3.2. Liver Histology

**Figure 1:**
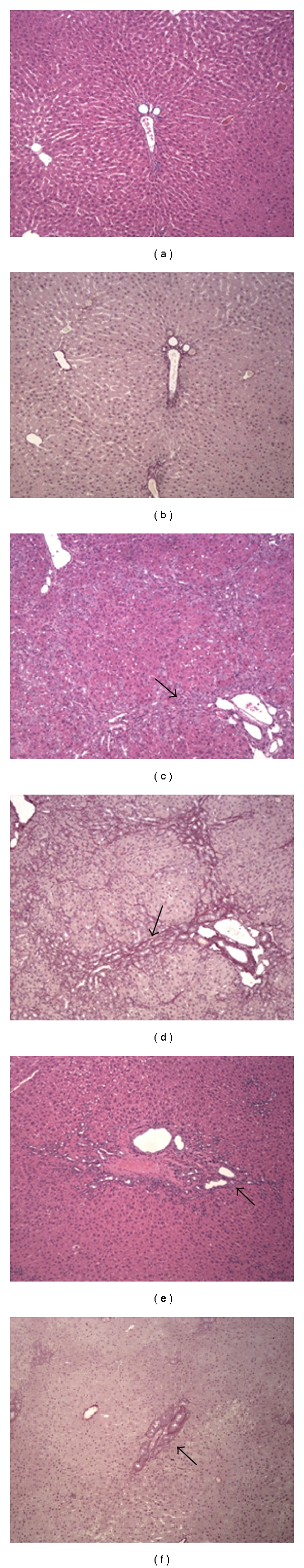
Photomicrographs of liver sections from rats (100x). Liver sections were stained with hematoxylin and eosin (a, c and e) or with picrosírius (b, d and f). (a) and (b): control rats (CO), showing normal architecture of liver parenchyma. (c) and (d): untreated rats with common bile duct obstruction (CBDL), showing regenerative nodules (arrow (c)) and fibrosis (arrow (d)). (e) and (f): Biliary obstructed rats treated with quercetin (CBDL + Q), showing the regeneration of liver parenchyma (arrow (e)) and light fibrosis (arrow (f)).

The histological analysis of liver tissue from the animals in the control group (CO) showed normal architecture of the parenchyma (Figures 1(a) and 1(b)). In animals with CBDL, there were a loss of normal architecture and a presence of regenerative nodules, cellular necrosis, (Figure 1(c)), and fibrosis (Figure 1(d)). In contrast, necrosis and fibrosis were minimal in animals from the group treated with quercetin (CBDL + Q) (Figures 1(e) and 1(f)). 

### 3.3. Parameters of Lipid Peroxidation, Antioxidant Enzyme Activities, and Total Nitrate Concentrations

The TBARS concentration was significantly increased in the CBDL rats, whereas this increase was inhibited by quercetin treatment. Lipid peroxidation was not modified by quercetin in normal rats (Table 2). 

We measured SOD enzymatic activity in erythrocytes and verified a reduction in enzymatic activity in the CBDL group compared to the CO and CO + Q and to the cirrhotic group treated with quercetin (CBDL + Q) (Table 2).

1. In the evaluation of the antioxidant enzymes GPx and CAT, both exhibited a significant reduction in the cirrhotic group CBDL. When animals were treated with quercetin, significantly increased enzyme activity, restoring values similar to those of the control group (Table 2).

Total liver nitrate concentration was significantly reduced in obstructed rats. Values were significantly increased in groups of quercetin-treated animals (Table 2). 

## 4. Discussion

Biliary cirrhosis is a chronic and diffuse hepatic disease that alters the intrahepatic or extrahepatic structure and the function of the biliary tree [27]. CBDL is used as an animal model of secondary biliary cirrhosis and, in four weeks, establishes cirrhosis with progressive and fatal damages to the liver. This model simulates the human disease, generating alterations from the inflammatory reaction caused by biliary ebb and the consequent disorganization of the parenchyma architecture, inflammatory and collagen depositions, and fibrosis formation [3].

Hepatic damage results in increased concentrations of serum AST, ALT, and ALP. Animals that underwent common bile duct ligation showed a significant increase in serial enzymes AST, ALT, and ALP, which are regarded as sensitive markers of hepatic damage and indicators of parenchymal and biliary tree injuries. Hepatic lesions and increases in these serial enzymes were also reported in several studies on bile duct obstruction [3, 5, 10].

Animals made cirrhotic by CBDL and treated with the antioxidant quercetin experienced restoration of the evaluated enzymes representing hepatic integrity. These results demonstrate that the flavonoid, due to its antioxidant potential, had a hepatoprotective function. Peres et al. have also reported quercetin treatment to reduce the high AST, ALT, and ALP levels present in bile duct obstruction-induced cirrhosis [6] and to effectively protect the liver against CCl4-induced hepatotoxicity [10]. In our study, CBDL was accompanied by regenerative nodules, necrosis, and centers of fibrosis. The cirrhotic animals treated with quercetin (CBDL + Q) exhibited this phenomenon to a lesser degree. These findings are in accordance with other studies that used quercetin or other antioxidant substances, such as rutin, N-acetylcysteine, and vitamins E and C, all of which decreased the severity of hepatic fibrosis [3, 5, 10, 17, 28]. 

When we evaluated the levels of total nitrates in the homogenized livers of the CBDL group, we observed a significant 52% reduction of these metabolites compared to the CO group (Table 2). In the CBDL + Q group, reduction of 26.4% was observed, suggesting that quercetin treatment inhibited the reduction of the metabolites activity due to an imbalance between vasoconstrictors and vasodilators. In the CO group, vasoconstrictors, such as endotelin-1, and vasodilators, such as nitric oxide (NO), are balanced in the liver. However, in an injured liver, the balance of these substances is lost. This imbalance characterizes the endotheliopathy of the cirrhotic liver, which has increased synthesis of endotelin-1. This phenomenon promotes vasoconstriction and increased intrahepatic resistance to blood flow, which provides an explanation for the low quantity of total nitrates present in this study [29]. Quercetin increases total liver nitrates due to its potential for scavenging free radicals, suggesting that it promotes increased circulation through the hepatic parenchyma.

Oxidative stress is defined as an imbalance between pro-oxidants and antioxidants; ROS-induced lipid peroxidation can occur either in overwhelmed scavenging systems (excessive production of ROS) or in impaired antioxidant systems. In cirrhotic animals, there is a considerable increase in liver lipoperoxidation due to the formation of ROS. When lipoperoxidation was evaluated, we observed an increase in TBARS in the erythrocytes of the CBDL animals compared to the CO rats. We suggest that the increase in this process is related to the increase in oxidative stress, which has already been shown in hepatic tissue by other studies [5, 6]. The increased oxidative stress in the CBDL model can be explained by endotoxemia and increased biliary acids, which can lead to an imbalance in the mitochondrial electron transport chain and can subsequently favor increased production of ROS [3, 30].

After quercetin treatment, animals from the CBDL group had TBARS values that were similar to those of the control group, indicating the antioxidant potential of this flavonoid. The reduction of lipoperoxidation in the hepatic tissue of the CBDL rats had already been demonstrated by the use of the antioxidant quercetin in the CBDL model [5, 6, 13].

Due to its antioxidant action, quercetin seems to protect the liver and ameliorate hepatic function; in a secondary pathway, it diminishes bacterial translocation from the gastrointestinal tract and, consequently, the severity of the disease [31].

The SOD enzyme is the cell's first line of defense against oxidative stress [32]. In the present study, we observed a reduction in SOD and CAT enzyme activity in the erythrocytes of cirrhotic animals compared to the other groups. Moreover, CBDL + Q animals maintained SOD enzyme activity similar to the animals of the CO group. Similar data were observed by Peres et al. in hepatic tissue treated with the flavonoid quercetin. These findings suggest that this flavonoid preserves SOD both in the hepatic tissue and in the erythrocytes of these animals [6]. Furthermore, the data reinforce the hypothesis that flavonoids, including quercetin, are potent sweepers of free radicals, such as hydroxyl radicals and superoxide anions, because the use of flavonoids results in an SOD activity similar to that of the CO group. We also observed a significant reduction of CAT activity in the erythrocytes from the cirrhotic group, possibly due to the excessive generation of H_2_O_2_ from dismutation of superoxide radical anions. In the animals treated with quercetin, CAT activity remained similar to that of the control group. 

GPx activity was significantly reduced in animals with CBDL, possibly due to increased superoxide anions and hydrogen peroxide, which presumably bind to the active site of the enzyme. Additionally, rats with CBDL lacked vitamin E, and selenium deficiency is known to cause depletion of tissue GPx activity, which makes tissue more vulnerable to oxidative damage [33]. The GPx enzyme is the primary catalytic cellular defense that protects cells and tissues against lipid peroxidation. A reduction of GPx activity was observed in homogenized liver and lung from cirrhotic animals, and this reduction was blocked after quercetin administration; in this study, we showed that a similar finding was observed in erythrocytes [3, 6, 30].

In summary, our data indicate that quercetin maintains the antioxidant defense system and reduces systemic oxidative damage, ductular proliferation, and fibrosis in biliary obstructed rats. Evaluation of blood during oxidative stress facilitates the monitoring of treatments and the evolution of disease. In this study, we were able to verify the same oxidative alterations reported previously by our group using blood samples instead of tissue homogenates, which were used in previous studies to evaluate oxidative stress on biliary cirrhosis [5, 13]. 

## Figures and Tables

**Table 1:** Effects of biliary obstruction determined by hepatic integrity tests.

Parameters	Experimental groups			
	CO	CO + Q	CBDL	CBDL + Q
AST (U/L)	95.67 ± 10.34	66.44 ± 2.26	510.6 ± 46.78^a^	146.9 ± 23.44
ALT (U/L)	65.78 ± 9.02	38.44 ± 2.51	127.5 ± 13.03^a^	52.8 ± 9.36
FA (U/L)	156.00 ± 16.62	143.33 ± 9.23	386.8 ± 27.71^a^	218.9 ± 42.73

CO: control; CBDL: common bile duct ligation; CO + Q and CBDL + Q: animals received a daily 50 mg/kg body i.p. injection of quercetin.

^a^Significant difference between the CBDL group and groups CO, CO + Q, and CBDL + Q; *P* < 0.05.

**Table 2:** Effects of quercetin (Q) on lipid peroxidation, antioxidant enzyme activities, and total nitrates on damage due to biliary obstruction.

Parameters	Experimental groups			
	CO	CO + Q	CBDL + Q	CBDL + Q
TBARS (nmol/mg Hb)	3.01 ± 0.22	2.46 ± 0.29	5.69 ± 0.23^a^	3.87 ± 0.06^b^
SOD (U-SOD/mg protein)	9.26 ± 0.55	8.26 ± 1.02	5.35 ± 0.53^a^	9.35 ± 0.46
CAT (pmol/mg protein)	0.1 ± 0.01	0.11 ± 0.01	0.05 ± 0.01^a^	0.09 ± 0.01
GPx (mmol/min/mg Hb)	3.45 ± 0.36	4.00 ± 0.61	2.15 ± 0.69^a^	4.63 ± 0.66
Nitrates (*μ*mol/L)	125.6 ± 18.1	110.5 ± 9.12	64.99 ± 5.67^a^	92.65 ± 5.75

CO: control; CBDL: common bile duct ligation; CO + Q and CBDL + Q: animals received a daily 50 mg/kg body i.p. injection of quercetin

Results represent the mean ± S.E.

^a^Significant difference between the CBDL group and the CO, CO + Q, and CBDL + Q groups; *P* < 0.05.

^b^Significant difference between the CBDL + Q group and the CO and CO + Q groups; *P* < 0.05.
